# Cholinergic System and NGF Receptors: Insights from the Brain of the Short-Lived Fish *Nothobranchius furzeri*

**DOI:** 10.3390/brainsci10060394

**Published:** 2020-06-20

**Authors:** Paolo de Girolamo, Adele Leggieri, Antonio Palladino, Carla Lucini, Chiara Attanasio, Livia D’Angelo

**Affiliations:** 1 Department Veterinary Medicine and Animal Production, University of Naples Federico II, Naples I-80137, Italy; adele.leggieri@unina.it (A.L.); lucini@unina.it (C.L.); chiara.attanasio@unina.it (C.A.); livia.dangelo@unina.it (L.D.); 2 CESMA—Centro Servizi metereologici e Tecnologici Avanzati, University of Naples Federico II, I-80126 Naples, Italy; a.palladino1986@gmail.com

**Keywords:** aging, fish, cholinergic system, NTRK1/NTRKA, p75/NGFR

## Abstract

Nerve growth factor (NGF) receptors are evolutionary conserved molecules, and in mammals are considered necessary for ensuring the survival of cholinergic neurons. The age-dependent regulation of NTRK1/NTRKA and p75/NGFR in mammalian brain results in a reduced response of the cholinergic neurons to neurotrophic factors and is thought to play a role in the pathogenesis of neurodegenerative diseases. Here, we study the age-dependent expression of NGF receptors (*NTRK1/NTRKA* and p75/*NGFR*) in the brain of the short-lived teleost fish *Nothobranchius furzeri*. We observed that *NTRK1/NTRKA* is more expressed than p75/NGFR in young and old animals, although both receptors do not show a significant age-dependent change. We then study the neuroanatomical organization of the cholinergic system, observing that cholinergic fibers project over the entire neuroaxis while cholinergic neurons appear restricted to few nuclei situated in the equivalent of mammalian subpallium, preoptic area and rostral reticular formation. Finally, our experiments do not confirm that *NTRK1/NTRKA* and *p75/NGFR* are expressed in cholinergic neuronal populations in the adult brain of *N. furzeri*. To our knowledge, this is the first study where NGF receptors have been analyzed in relation to the cholinergic system in a fish species along with their age-dependent modulation. We observed differences between mammals and fish, which make the African turquoise killifish an attractive model to further investigate the fish specific NGF receptors regulation.

## 1. Introduction

The brain of teleost fish has received much attention in the last decades, with regards to basic and applied neuroscientific research [1,2]. Among fish species, the most well studied model organism is *Danio rerio*, commonly known as zebrafish, widely employed in genetics research, neurophenotyping and central nervous system (CNS) drug screening, as well as in modeling complex neurological and psychiatric disorders [3]. On the other hand, the process of brain aging in the teleost has received much less attention so far [4,5]. For brain aging studies, the African turquoise killifish, *Nothobranchius furzeri,* has emerged as a powerful model, due to its natural lifespan ranging between 4 and 9 months, related ageing hallmarks and the available approaches for experimentally modulating the lifespan [6,7]. In the course of aging, *N. furzeri* shows reduced learning performances, paralleled by gliosis and reduced adult neurogenesis [4]. In addition, brain displays evolutionary conserved miRNA regulation [8,9].

Recently, our group has dedicated a lot of research efforts to identify the pattern of expression of all neurotrophins in the brain of the African turquoise killifish [9,10,11,12,13]. Neurotrophins constitute a family of evolutionary well-conserved molecules, and act in multiple context-dependent biological functions, including neuronal cell death and survival, neurite outgrowth and neuronal differentiation [14]. They play pleiotropic as well as fundamental roles in the central nervous system (CNS) of vertebrates and are thus deeply involved in several neurodegenerative conditions [15,16]. However, their actions depend upon the binding to different classes of receptors, tyrosin kinase receptors (NTRKs), and member of the tumor necrosis factor, commonly named p75/NGFR [14].

In teleost fish lineage, specific NTRK receptors gene duplication has occurred [17], resulting in five genes encoding for NTRK-receptors. Differently from NTRK2/NTRKB and NTRK3/NTRKC, which exist in two isoforms, the duplicated NTRK1/NTRKA-gene was lost early in the fish lineage and only one isoform is available in the fish genome [18]. During fish development, *NTRK1/NTRKA* appears 24 h post fertilization in cranial nerves and rostral hindbrain [18]. Immunohistochemical studies have documented that protein encoding NTRK1/NTRKA is distributed in the brain of adult *N. furzeri* [19] and zebrafish [20] and it is considered a marker of crypt cells in the olfactory organ of fish [21,22] where it seems to mediate the immune antiviral response [23]. The teleost *p75/NGFR* gene family has been poorly characterized so far. This receptor is one of the molecular components of the Nogo/NgR signaling pathway, which is known to be conserved in zebrafish [24,25]. Genes orthologous to those encoding the three mammalian ligands, NgR and the co-receptor p75, are represented in the fish genome. Numerous killifish species, including *N. furzeri*, possess only one copy of *p75/NGFR* orthologs. Differently, in the zebrafish genome, the most accredited hypothesis supports the persistence of at least two isoforms, *p75/NGFR1a* and *p75/NGFR1b* [26]. In zebrafish, genes encoding p75/NGFR show a striking similarity in the spatial and temporal expression patterns to that observed in mammalian species [27,28,29,30]. 

The balance between NTRK receptors and p75/NGFR is crucial to the functional outcome of neurotrophin binding; sufficient amounts of activated NTRKs, for example, can suppress apoptotic pathways activated by p75/NGFR [31,32]. p75/NGFR, when complexed with NTRK1/NTRKA, increases NGF signaling through NTRK1/NTRKA to enhance survival and neurite outgrowth [33]. In mammals, NGF and related receptors are necessary for ensuring the survival of cholinergic neurons [34,35]. The expression of NTRK1/NTRKA mRNA appears to be restricted to neurons of the basal forebrain and caudate-putamen, with features of cholinergic cells and to magnocellular neurons of several brainstem nuclei [36]. Similarly, *p75/NGFR* expression is colocalized exclusively with cholinergic neurons in the basal forebrain, and it is among the earliest cholinergic markers expressed during development [37]. During zebrafish development, *p75/NGFR* is localized in the cholinergic cells of the ventral basal forebrain, in mid- and hindbrain nuclei, cranial ganglia, the region of the locus coeruleus and in dorsal root ganglia [38]. In mammalian brain, the age-dependent downregulation of *NTRK1/NTRKA* and *p75/NGFR* results in a reduced response of the cholinergic neurons to neurotrophic factors and is thought to play a role in the pathogenesis of neurodegenerative diseases [39,40,41,42].

Acetylcholine is synthesized from choline and acetyl CoA by the transferase enzyme choline acetyl-transferase (ChaT), a specific marker commonly used as a reliable indicator of cholinergic neurons [43]. The cholinergic system is an important ubiquitous system in vertebrate brains, and it is implicated in processes such as the modulation of behavior, learning and memory, the sleep-wakefulness cycle and superior cognitive functions [44]. Cholinergic neurons and fibers are localized in homologous brain areas of non-mammalian vertebrates, reinforcing the idea of an evolutionary conservation of the system, although in each group species-specific features have been reported [45,46,47,48,49,50,51,52,53].

Based on this evidence, here we propose to investigate the age-dependent regulation of *NTRK1/NTRKA* and *p75/NGFR* in the brain of *N. furzeri*, their pattern of expression, and evaluate if cholinergic neurons are the main source of *NTRK1/NTRKA* and *p75/NGFR* in the adult brain of the killifish. To accomplish this aim, we therefore also studied the neuroanatomical organization of the cholinergic system in adult specimens of African turquoise killifish. 

## 2. Materials and Methods

### 2.1. Animals and Tissue Sampling

The experimental protocols were approved by the Animal Welfare Body of University of Naples Federico II (2015/0023947). Experiments were performed on the *Nothobranchius furzeri* MZM–04/10 strain of both sexes. Animals’ maintenance was performed as previously described [54]. Young (5 weeks post hatching), adult (14 weeks post hatching) and old (27 weeks post hatching) animals were euthanized with a 0.1% solution of ethyl 3-aminobenzoate methane sulfonate (MS—222; Sigma, St. Louis, MO, USA; A-5040). In order to avoid the effect of circadian rhythms and feeding, all animals were suppressed around 10 a.m. For RNA extraction, fish were decapitated, brains were rapidly dissected, kept in sterile tubes (Eppendorf) with 500 µL of RNAlater (Qiagen, Hilden, Germany), and stored at 4 °C until RNA extraction.

For fluorescence *in situ* hybridization, combined fluorescence *in situ* hybridization and immunofluorescence (IF), and light immunohistochemistry, animals were decapitated, heads were rapidly excised and fixed in a sterile solution of paraformaldehyde (PFA) 4% in phosphate buffered saline (PBS) overnight (ON). Successively, brains were dissected and incubated in 30% sucrose solution ON, 4 °C, and then in 20% sucrose solution ON, 4 °C; finally, they were embedded in a cryomounting medium and stored at −80 °C. Serial transversal sections of 14 µm thickness were cut with a Leica cryostat (Leica, Deerfield, IL, USA).

For light immunohistochemistry, 5 heads of adult animals were collected and fixed in solution of PFA) 4%, processed for paraffin embedding, and serial transversal and sagittal sections of 7 µm thickness were obtained. 

### 2.2. RNA Isolation and cDNA Synthesis

Tissues were taken out of RNAlater and cleaned with sterile pipettes. *N. furzeri* total RNA was isolated from 12 animals with QIAzol (Qiagen), as previously described [10]. Homogenization was performed using a TissueLyzer II (Qiagen) at 20 Hz for 2–3 × 1 min. Total RNA was then quantitated with Eppendorf BioPhotometer (Hamburg, Germany). Five hundred nanograms of each sample was retrotranscribed to cDNA in a 20 µL volume, using the QuantiTect^®^ Reverse Transcription Kit (Qiagen), following the supplier’s protocol. Newly synthetized cDNAs were then diluted to a final volume of 200 µL with ultra-pure sterile water to an approximate final cDNA concentration of 40 ng/µL.

### 2.3. Quantitative Real Time-PCR

Primers were designed with the Primer3 tool. All reactions were performed in triplicate and negative control (water) was always included. Reactions were performed in a 20 µL volume containing 1 µL of diluted cDNA, using BrightGreen 2X qPCR MasterMix kit (abm^®^, Richmond, BC, Canada) following the manufacturer’s instructions. After the initial heat activation for 2 min at 95 °C, a 2-step cycling was run (denaturation 5 s at 95 °C, combined annealing/extension 10 s at 60 °C) for 35 cycles. Primers were the following: 

*NTRK1/NTRKA*: forward 5′-ATGGTGCAATTGGACATTGA-3′; reverse 5′-TACAGCCAGGTGATGTTTGG-3′;

*p75/NGFR*: forward 5′-ACCGTGTCGAGACTCACAGA-3′; reverse 5′-TGTAGGGCTGTGCACTGTGT-3′.

### 2.4. Statistical Analysis

Expression levels of NTRK1/NTRKA and p75/NGFR mRNAs were analyzed by the ΔΔCt method and normalized to the housekeeping gene TATA box binding protein (TBP): forward 5′-CGGTTGGAGGGTTTAGTCCT-3′; reverse 5′-GCAAGACGATTCTGGGTTTG-3′). Fold changes represent the difference in expression levels of the two receptors between the time points analyzed, respectively with young and old age TATA-binding protein (TBP) cDNAs. The relative ΔΔ curve threshold was built on fold changes values and the *p*-value was <0.01. Statistical analysis of quantitative real-time data was done by an unpaired two-tailed *t* test and Pearson correlation by using GraphPad Prism 8 (GraphPad Software, San Diego, CA, USA).

### 2.5. In Vitro Transcription and Probe Synthesis

mRNA probes to identify NGF mRNA receptors were synthetized by in vitro transcription (IVT) using the MAXIscript™ SP6/T7 in vitro transcription kit (Catalogue number AM1312, Invitrogen by Thermo Fisher Scientific, Carlsbad, CA. USA) and following the manufacturer’s instructions. One microgram of the DNA template was transcribed to RNA in 20 µL volume reaction, using primers associated with the T7 promoter sequence (*NTRK1/NTRKA* forward 5′-ATGGATGGAAACCCTGAGCC-3′; *NTRK1/NTRKA* T7 reverse 5′-GGTAATACGACTCACTATAGG_GTGTGTTTGAAGCTGCTCGA-GTTGATGTGGGTCGGCTTA-3′; *p75/NGFR* forward 5′-TCGATGAAGAGCCATGTTTG-3′, *p75/NGFR T7* reverse 5′-GGTAATACGACTCACTATAGG_GCCTCATCTGGGAGTGGTAA-3′) and a DIG RNA Labeling Mix, 10× concentration (Roche, Basel, Switzerland) containing digoxigenin labeled uracil. After the IVT reaction, the product was briefly centrifuged and incubated at 37 °C for 1 h. Then 1 µL of turbo DNase 1 was added, the sample was mixed well and incubated for 15 min at 37 °C. One microliter of EDTA 0.5 M was added to stop the reaction. Reaction product was analyzed by gel electrophoresis and quantified.

### 2.6. Fluorescence In Situ Hybridization

In situ hybridization was performed on cryosections using sterile solutions and materials. Sections were dried for 2 h at room temperature (RT), well washed in 1 × DEPC/PBS and treated with 10 µg/µL Proteinase K (Sigma–Aldrich), diluted 1:200 in DEPC/PBS for 10 min. Sections were then washed twice in 2 mg/mL glycine, 5 min each to inactivate proteinase K. Sections were post fixed in PFA 4% for 20 min and well washed in 1× DEPC/PBS at RT. Thereafter, the prehybridization was carried out in a hybridization solution (HB) containing 50% formamide, 25% 20× SSC, 50 µg/mL Heparin, 10 µg/mL yeast RNA, 0.1% Tween 20 and 0.92% citric acid at 52 °C for 1 h. All probes were denatured for 10 min at 80 °C and sections were then incubated, in HB containing riboprobes concentration of 500 pg/µL, ON at 52 °C. Post-hybridization washes were carried out at 52 °C as follows: 2 × 20 min in 1× SSC, 2 × 10 min in 0.5× SSC and then in 1× DEPC/PBS at RT. Sections were blocked in the blocking solution (BS) containing 10% normal sheep serum heat inactivated and 0.5% blocking reagent (Roche, Basel, Switzerland) for 1 h at RT. Later, sections were incubated in a 1:2000 dilution of anti-digoxigenin Fab fragments conjugated with alkaline phosphatase (Roche) in BS, 2 h at RT. Sections were well washed in 1× DEPC/PBS. The chromogenic reaction was carried out by using Fast Red tablets (Sigma-Aldrich) in Tris buffer and incubating the slides at RT in the dark and was observed every 20 min until signal detection. Finally, sections were washed in 1× DEPC/PBS at RT and mounted with the Fluoreshield mounting medium with DAPI as counterstaining for the nuclei.

### 2.7. Combined Fluorescence In Situ Hybridization with Immunofluorescence

After the detection of the ISH chromogenic reaction, sections of adult animals were well washed in DEPC/PBS, and incubated at RT for 1 h with a blocking serum (normal goat serum 1:5 in PBS containing 0.1% Triton X-100, Sigma) and subsequently with primary antiserum (Anti-ChaT, cat. #AB143, Merck Millipore, Burlington, MA. USA) 1:100, at 4 °C ON. DEPC/PBS washes preceded the incubation with the secondary antibodies: goat anti-rabbit IgG (H+L) Alexa fluor™ Plus 488 (1:1000, Invitrogen by Thermo Fisher Scientific, ref. A32731).

### 2.8. Light Immunohistochemistry

Immunohistochemistry was conducted on cryo- and paraffin embedded sections according to previous protocols [54]. Cryosections were dried for 2 h at RT. For paraffin sections, slides were dewaxed and hydrated. All sections were rinsed in distilled water for 5 min. Antigen retrieval was performed by microwave oven treating (10 min, 750 W) with citrate tampon (0.01 M, pH 6.00). Endogenous peroxidase activity was blocked with 3% H_2_O_2_ treatment (20 min, RT). After three washes (5 min, RT) with PBS, slides were pre-incubated with normal goat serum (NGS), 1:5 diluted in PBS for 30 min in humid chamber, RT and then incubated with rabbit Anti-ChaT (Merck Millipore, cat. #AB143), 1:1000 at 4 °C ON. After the washes in PBS, sections were incubated at RT for 30 min with Dako EnVision + System − HRP labeled polymer. The immunoreactivity of the cells was visualized using a freshly prepared solutions of 3,3′—diaminobenzidine tetrahydrochlride (Sigma Aldrich) activated with a solution of 0.03% H_2_O_2_, after which the sections were mounted.

### 2.9. Microscopy

Images were analyzed by Leica—DM6B (Leica, Wetzlar, Germany) and processed with LasX software (Leica, Microsystems, Wetzlar, Germany). The digital raw images were optimized for image resolution, contrast, evenness of illumination and background using Adobe Photoshop CC 2018 (Adobe Systems, San Jose, CA, USA). Anatomical structures were identified according to the adult *N. furzeri* brain atlas [55].

## 3. Results

### 3.1. Pattern of Distribution of ChaT in the Adult Brain of N. furzeri

**Figure 1:**
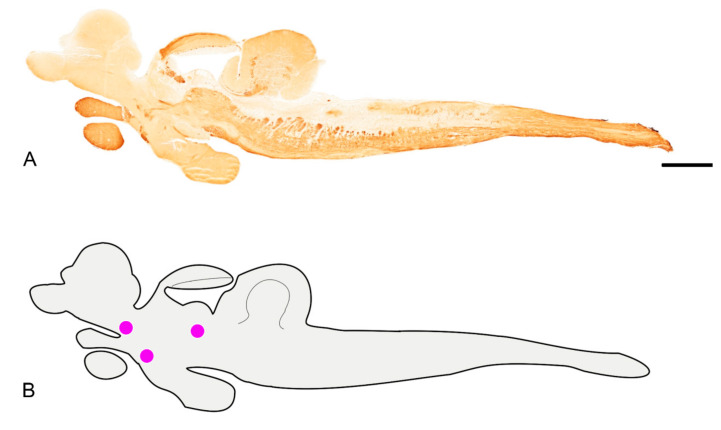
Overview of the brain of adult *N. furzeri*. (**A**) Sagittal section of the whole brain showing diffuse neuronal projections and very few groups of cholinergic nuclei. (**B**). Schematic view of sagittal section A showing in violet the identified groups of cholinergic nuclei (Ch-1, Ch-2 and Ch-3). Scale bar = 2.5 µm.

We characterized the neuroanatomical organization of the cholinergic system in the adult N. furzeri, an overview of the immunohistochemical distribution is given in Figure 1A,B.

*Forebrain*

**Figure 2:**
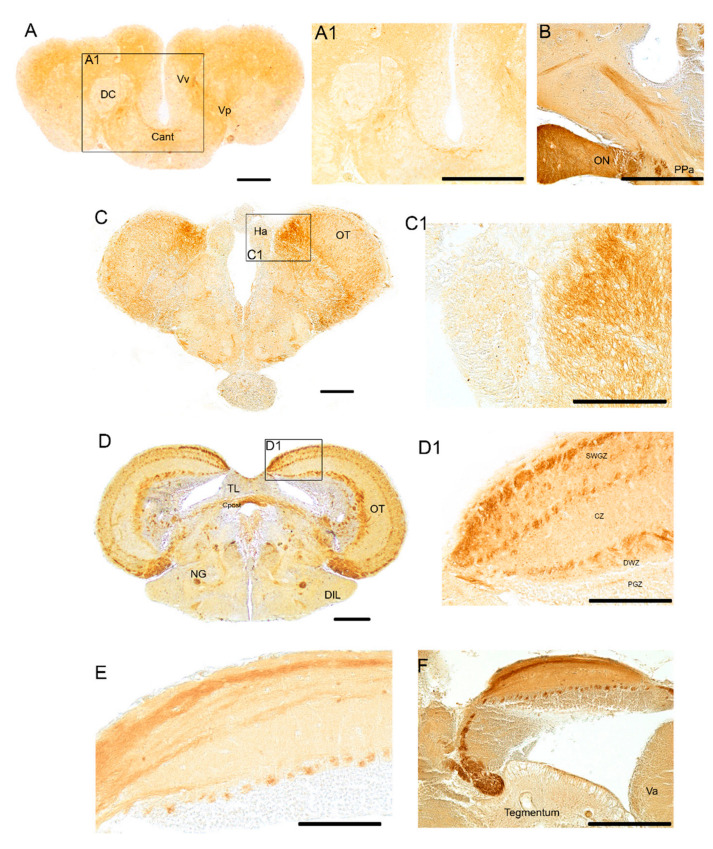
Forebrain and midbrain of adult *N. furzeri*. (**A**). Transverse section of the forebrain with numerous immunopositive fibers over the telencephalon and in the varicose fibers of the anterior commissure. (**A1**). Higher magnification of the rectangle in A showing varicose fibers of the anterior commissure. (**B)**. Sagittal section of forebrain showing a group consisting of few positive neurons in the preoptic area in the rostral diencephalon and numerous fibers projecting toward the telencephalon. (**C**). Transverse section of the pretectal region and in the most rostral part of optic tect, epithalamus, thalamus and rostral part of hypothalamus depicting numerous widespread immunopositive fibers (**C1**). Higher magnification of the rectangle in C showing positive fibers of OT and faintly labeled cells in the ventro-lateral part of habenular nucleus. (**D**). Transverse section of caudal diencephalon/anterior midbrain displaying immunoreactivity to ChaT in fibers, displaced along the dorsal margin of longitudinal tori and in the posterior commissure; wide distribution of positive fibers in the whole posterior thalamic area/anterior midbrain tegmentum and posterior commissure. (**D1**). Higher magnification of the rectangle in D showing immunoreactivity in fibers of the deep white zone and superficial white and gray zone of OT. (**E**). Sagittal sections showing strong immunoreactivity in fibers of the deep white zone (resembling glomeruli) and superficial white and gray zone of OT. (**F**). Sagittal sections showing strong immunoreactivity in fibers of OT and ascending fibers from the pretectal region. Abbreviations: anterior commissure (Cant); central zone of dorsal telencephalon (DC); diffuse inferior lobe of hypothalamus (DIL); habenular nucleus (Ha); glomerular nucleus (NG); optic nerve (ON); optic tect (OT); layers of OT: periventricular grey zone (PGZ); deep white zone (DWZ); central zone (CZ); superficial white and gray zone (SWGZ).anterior preoptic nucleus (PPa); longitudinal tori (TL); posterior zone of ventral telencephalon (Vp); valvular of cerebellum (Va); ventral telencephalon (Vv). Scale bars = A, C, D 2.5 µm; A1, B, D1, E, F = 50 µm; C1 = 100 µm.

Numerous immunopositive fibers were distributed in the olfactory bulbs, and in the telencephalon (Figure 2A,A1), mostly in the ventral (Vv) and posterior (Vp) divisions of ventral telencephalon. Intense immunoreactivity was observed in the varicose fibers of the anterior commissure (Figure 2A,A1). A group of a few positive neurons was observed in the ventral telencephalon and in the preoptic area (Figure 2B) in the rostral diencephalon. We named these two cholinergic groups respectively Ch-1 and Ch-2, according to the mammalian brain homologies of mammalian septum and basal forebrain cholinergic neurons [56,57]. Intense immunoreactivity was observed in the lateral forebrain bundle, faintly stained appeared the cells in the ventro-lateral part of habenular nucleus, and in the whole thalamic area (Figure 2C); Intense staining was detected in fibers in the preglomerular nucleus, in proximity of the ventricle (Figure 2C) and in neuronal projections of the central pretectal nucleus (Figure 2C,C1).

*Midbrain*

An intense immunoreactive bundle of neuronal projections ascending toward the optic tect (OT) was observed in its most cranial part (Figure 1A and Figure 2D–F). In the OT, immunoreactivity was seen in fibers of the deep white zone and superficial white and gray zone (Figure 2D,E).

**Figure 3:**
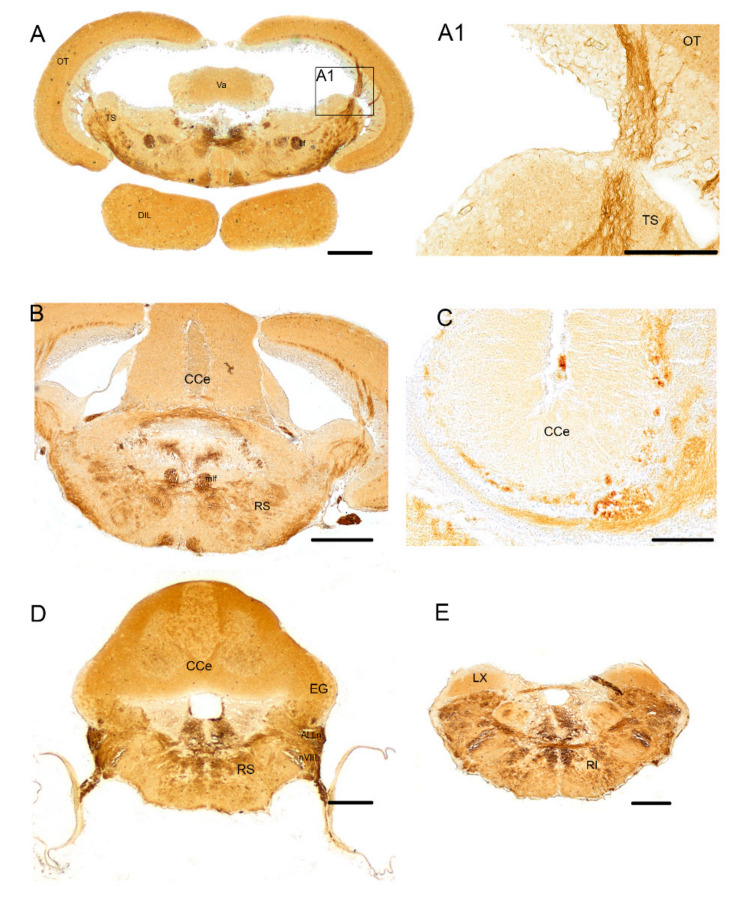
Midbrain and hindbrain of adult *N. furzeri*. (**A**). Transverse section of midbrain showing strongly immunoreactive fibers in the lateral and medial forebrain bundle, central griseum, ansulate commissure, cruciate tecto-bulbar tract and the most cranial part of semicircular tori projecting towards the OT. (**A1**). Higher magnification of rectangle in A showing ascending fibers from the tegmentum to OT. (**B**). Transverse section of rostral hindbrain showing a bundle of immunoreactive fibers running over the body of cerebellum and a group of few positive neurons in the rostral reticular formation. (**C**). Sagittal section of cerebellum showing intense immunoreactive fibers running from the valvula to the body of the cerebellum. (**D**). Transverse section of the hindbrain displaying a group consisting of positive neurons in the rostral reticular formation together with densely immunopositive fibers and moderately positive fibers in the ventral rhombencephalic commissure, and the caudal part of the cruciate tecto-bulbar tract, and more laterally ChaT immunoreactivity in neuronal projections from the octavolateral area running over the inner ear. (**E**). Transverse section of medulla oblongata at the spinal cord junction. Intensely immunopositive fibers were observed in the most caudal part of medulla oblongata, at the margin with spinal cord displaying a dense mesh of positive fibers over the entire medulla oblongata, including caudal reticular formation. Any staining was observed in the caudal part of the vagal lobe, displaced dorsally. Abbreviations: lateral forebrain bundle (llf); medial forebrain bundle (llf); semicircular tori (TS); body of cerebellum (CCe); granular eminentia of cerebellum (EG); inferior reticular formation (RI); superior reticular formation (RS); anterior part of nerve of lateral line (nALL); octavolateral nucleus (nVIII); vagal lobe (LX). Scale bars = A, B, D, E = 2.5 µm; A1, C = 100 µm.

Immunoreactivity to ChaT was seen in fibers, displaced along the dorsal margin of longitudinal tori and in the posterior commissure (Figure 2D). Wide distribution of positive fibers was seen in the posterior thalamic area/anterior midbrain tegmentum. More ventrally, few positive neurons were detected in the diffuse inferior lobe of the hypothalamus (Figure 1A and Figure 2D). Strongly immunoreactive fibers were seen in the lateral forebrain bundle (Figure 3A), medial forebrain bundle, central griseum (Figure 3A), ansulate commissure, cruciate tecto-bulbar tract and the semicircular tori projecting towards the optic tect (Figure 3A,B).

*Hindbrain*

In the cerebellum, a bundle of immunoreactive fibers running over the valvula and body of cerebellum were intensely immunoreactive to ChaT (Figure 1A and B,C). Widespread positive fibers were detected in the rostral, intermediate and caudal reticular formation of the medulla oblongata (Figure 1A and Figure 3B,D,F), and moderately dense fibers in the ventral rhombencephalic commissure, and caudal part of the tectobulbar tract (Figure 3D,F). A group of a few positive neurons was observed in the nucleus of rostral reticular formation (Figure 1A and Figure 3B). We named this group Ch-3, according to the mammalian brain homologous of the pedunculopontine nucleus and dorsolateral tegmental group [58,59]. Along the ventricle, a mesh of ChaT positive fibers was detected (Figure 1A and Figure 3D). Intense immunoreactivity was seen in neuronal projections from the octavolateral area running over the inner ear (Figure 3D). With the exception of the most caudal part of the vagal lobe, which appears devoid of immunoreactive neuronal projections, a dense mesh of positive fibers was appreciable over the entire medulla oblongata (Figure 3F).

### 3.2. Age-Dependent Expression of NTRK1/NTRKA and p75/NGFR in the Brain of N. furzeri

**Figure 4:**
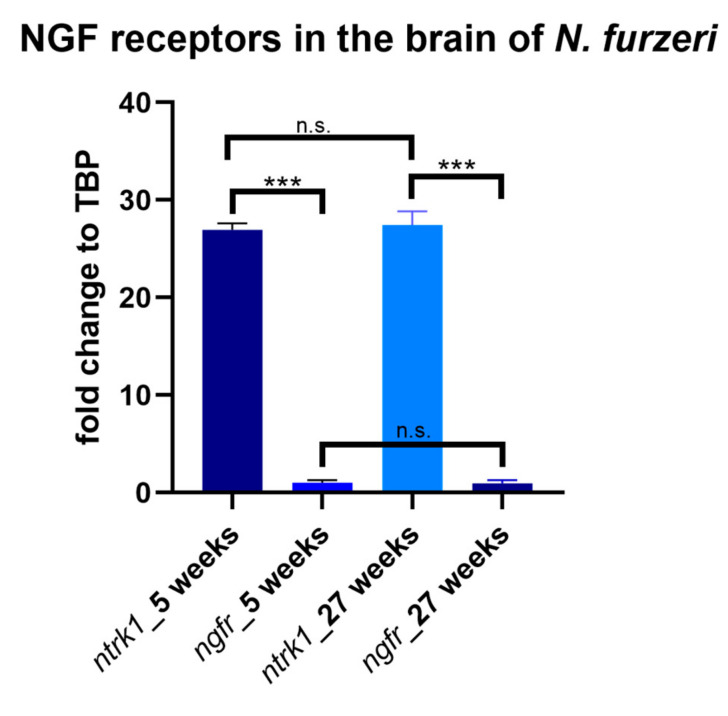
Age-dependent expression of *NTRK1/NTRKA* and p75/*NGFR* in the brain of *N. furzeri*. Either *NTRK1/NTRKA* and p75/*NGFR* display unchanged expression levels between young and old stages (*NTRK1/NTRKA p* value = 0.27; *ngfr p* value = 0.54). However, comparing the expression of the two receptors, *ntrk1* is significantly more expressed than p75/*NGFR*, in both 5 (*p* value ≤ 0.0001) and 27 (*p* value ≤ 0.0001) weeks post hatching animals. *** *p* value ≤ 0.0001

NTRK1/NTRKA and p75/NGFR mRNAs are both expressed in the brain of *N. furzeri* with differences in their expression levels. Quantitative measurements revealed that either *NTRK1/NTRKA* and *p75/NGFR* display unchanged expression levels in the brain at 5 and 27 weeks post hatching (*NTRK1/NTRKA p* value = 0.27; p75/NGFR *p* value = 0.54; Figure 4). A statistically significant difference was observed between the expression levels of *NTRK1/NTRKA* and *p75/NGFR* at 5 weeks (*p* value ≤ 0.0001) and at 27 weeks (*p* value ≤ 0.0001); Figure 4). We furthermore observed a positive linear correlation between the two NGF receptors, at the two age stages examined (5 weeks r = 0.9986; 27 weeks r = 0.9946).

The quantitative measurements matched with our morphological observations, with *NTRK1/NTRKA* more abundantly expressed in the brain of *N. furzeri* compared to *p75/NGFR*, either at young and old stages. The sense probe for NTRK1/NTRKA is shown in Figure S1.

**Figure 5:**
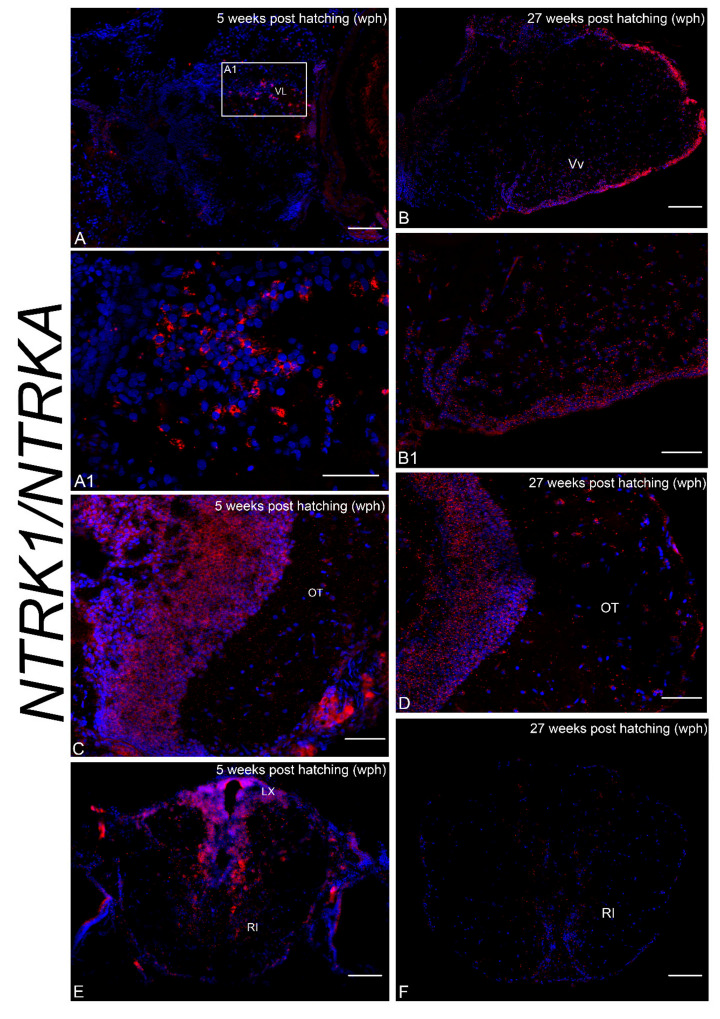
NTRK1/NTRKA mRNA in the brain of young and old *N. furzeri*. (**A**). Transverse section of caudal telencephalon of young animals displaying intense labeling in neurons of the lateral thalamic nucleus. (**A1**). Higher magnification of the rectangle in A showing positive neurons of the lateral thalamic nucleus. (**B**). Transverse section of caudal telencephalon of old animals displaying intense labeling in neurons of the ventral zone of the telencephalon. (**B1**). Higher magnification of B showing positive neurons of the ventral zone of the telencephalon. (**C,D**) Numerous positive cells in the periventricular gray zone of the OT and very few cells in the more superficial layers of young (**C**) and old (**D**) animals. (**E,F**). Overview of the hindbrain of young and old animals showing *ntrk1* expressing neurons in the vagal lobe, along the caudal part of the rhomboencephalic ventricle and in the caudal reticular formation of young animals, and faint labeling along the ventricle and in the reticular formation of old animals. Abbreviations: ventro-lateral thalamic nucleus (VL). Scale bars = A, B, F = 2.5 µm; B1, D, E = 50 µm; A1, C = 100 µm.

In the forebrain, *NTRK1/NTRKA* expressing neurons were observed in the caudal telencephalon, pretectal nucleus, anterior and lateral (Figure 5A,A1) thalamic nuclei of young animals, and in the ventral zone of the telencephalon (Figure 5B,B1) of old animals. More caudally, positive neurons were observed in the diffuse inferior lobe of hypothalamus at the two age stages analyzed. In the midbrain, numerous positive cells were observed in the periventricular gray zone of the OT and very few sparse cells in the more superficial layers of young and old animals (Figure 5C,D). Very faint signal probe was observed in the semicircular tori of the tegmentum and lateral nucleus of cerebellar valvular in the young animals. Caudally, in the hindbrain neurons expressing *NTRK1/NTRKA* were observed in the vagal lobe, along the caudal part of the rhomboencephalic ventricle and dispersed in the caudal reticular formation (Figure 5E) of young animals, whereas in the brain of old animals NTRK1/NTRKA mRNA expression was faintly observed along the ventricle and in the reticular formation (Figure 5F).

The expression of p75/NGFR mRNA was considerably less widespread compared to *NTRK1/NTRKA* at the two analyzed age stages. 

**Figure 6:**
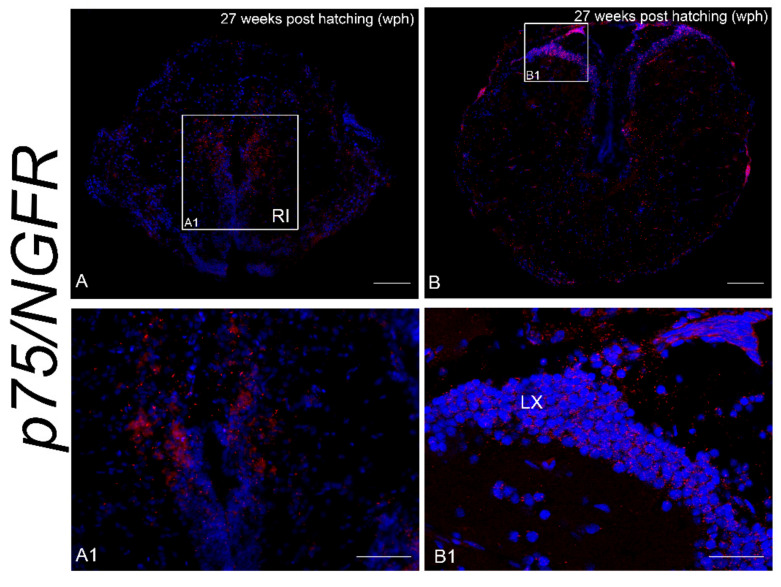
p75/NGFR mRNA in the brain of young and old *N. furzeri*. (**A**). Transverse section of medulla oblongata of young animals showing intense staining in neurons of the caudal reticular formation, around the caudal part of ventricle/anterior margin of ependymal canal. (**A1**) Higher magnification of rectangle in (**A**) depicting *p75/NGFR* expressing neurons located along the rhomboencephalic ventricle/rostral part of ependymal canal. (**B**) Transverse section of medulla oblongata of old animals showing labeling in neurons of the vagal lobe and along the ventricle. (**B1**) Higher magnification of rectangle in (**B**) depicting p75/NGFR expressing neurons in the vagal lobe. Scale bars = A, B = 2.5 µm; A1 = 50 µm; B1 = 100 µm.

p75/NGFR mRNA was faintly seen in the granular and molecular layers of the body of cerebellum in young animals. Both in young and old animals, intense staining was observed in neurons of the caudal reticular formation in the medulla oblongata, around the caudal part of ventricle/anterior margin of ependymal canal (Figure 6A,A1,B) and in the vagal lobe (Figure 6B,B1).

### 3.3. NTRK1/NTRKA and p75/NGFR Are Not Colocalized with ChaT

**Figure 7:**
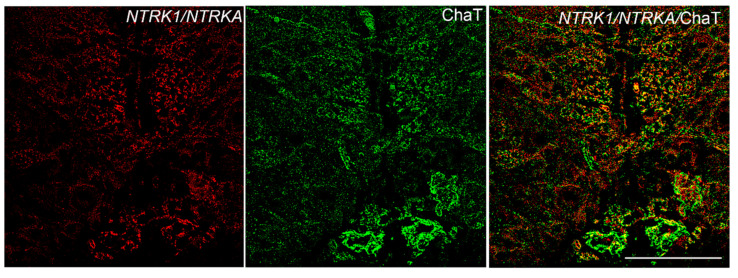
Transverse section of rostral reticular formation of adult *N. furzeri* showing staining of *NTRK1/NTRKA* (red), ChaT (green) and merged. Any neuronal co-staining was observed in the merged figure, only few cholinergic fibers appeared to contact some labeled *NTRK1/NTRKA* neurons. Scale bar = 50 µm.

We then conducted a combined in situ hybridization and immunohistochemistry to evaluate whether NTRK1/NTRKA and p75/NGFR expressing neurons showed co-localization with ChaT neurons in the adult or their neuronal projections. Any neuronal co-staining was observed between NTRK1/NTRKA and ChaT nor p75/NGFR and ChaT in the adult brain of *N. furzeri*. Only few cholinergic fibers appear to contact some labeled *NTRK1/NTRKA* expressing neurons in the rostral reticular formation (Figure 7).

## 4. Discussion

In this study, we investigated the neuroanatomical organization of the cholinergic system and we questioned whether cholinergic neurons were also *NTRK1/NTRKA* and *p75/NGFR* expressing neurons in the brain of *N. furzeri*. Cholinergic neurons are distributed in the nervous system of all vertebrates and are involved in the control of motor functions as well as in complex cognitive functions and behaviors. Cholinergic systems are also associated to age-dependent neurodegeneration [58,59,60], also caused by an imbalance of neurotrophins and related receptors [61]. 

### 4.1. Organization of the Cholinergic System in N. furzeri

Cholinergic system organization, based on the immunodetection of choline acetyltransferase, the enzyme that catalyzes the synthesis of acetylcholine in cholinergic neurons, has been widely investigated in all vertebrates mammals [61,62,63,64,65,66,67,68,69]; birds [70,71]; reptiles [72,73,74]; amphibians [75,76] and fish [45,46,47,48,49,50,51,52,53,77,78,79,80,81]. The pattern observed in *N. furzeri* revealed wide neuronal projections distribution throughout the entire neuroaxis, and very few cholinergic neurons restricted to the ventral telencephalon, preoptic area and diffuse inferior lobe of hypothalamus. According to the homologies with mammals, we defined three relevant groups of cholinergic neurons in *N. furzeri*. (a) Ch-1: ChaT immunoreactive cells in the ventral telencephalon, considered the fish subpallium, are likely homologous to cholinergic septal neuron populations in tetrapods and represent a well-conserved cell group found in fishes, amphibians, reptiles, birds and mammals [81]; (b) Ch-2: the cholinergic cells identified in the preoptic area, also documented in other teleosts [47,48,50,53,81], correspond functionally to the mammalian basal forebrain cholinergic groups [59]; (c) Ch-3: the cholinergic cells observed in the rostral reticular nucleus, are equivalent to the pedunculopontine nucleus and dorsolateral tegmental group of mammals. In addition, we demonstrated evidence of cholinergic fibers in the olfactory bulbs, similar to zebrafish [80], but not documented in the forebrain of other fish species [81]. We observed a diffuse network of varicose ChaT-positive fibers innervating the mitral cell/glomerular layer. Finally, we found ChaT positive neurons in the diffuse inferior lobe of the hypothalamus but not in other hypothalamic regions. In many fish species, the hypothalamus is free of cholinergic cells and displays only positive neuronal projections. In zebrafish, for example, the ChaT immunopositive input to the hypothalamic orexin cluster was observed [48].

### 4.2. Age-Associated Regulation of Nerve Growth Factor Receptors and Comparison of Their Neuroanatomical Expression

We further investigated the age-related changes of the two nerve growth factor receptors, NTRK1/NTRKA and p75/NGFR, in the brain of the African turquoise killifish, and their co-localization in cholinergic neurons. We report that the genes encoding *NTRK1/NTRKA* and *p75/NGFR* are not duplicated in the studied model species. This makes the African turquoise killifish a powerful model for further translational studies. Available transcriptomic data on the *NTRK1/NTRKA* [82] document low expression levels in the brain of specimens of the long-lived strain, without age-dependent regulation, whereas *p75/NGFR* seems to be overexpressed in the brain of old animals. We did not observe any statistically significant age-dependent modulation neither for *NTRK1/NTRKA* nor *p75/NGFR* in the brain of *N. furzeri*. Our results demonstrate that there is no increase of *NTRK1/NTRKA* nor a decrease of *p75/NGFR* in the whole brain of old animals. In mammals, including humans, NTRK1/NTRKA mRNA is down-regulated in the course of aging and neurodegenerative processes [83,84,85,86]. This decreases the amount of NTRK1/NTRKA protein destined for anterograde transport to basal forebrain cholinergic neurons distal axon terminals [85]. Very interestingly, NTRK1/NTRKA protein levels are reduced in the cortex of Alzheimer’s disease patients, while many studies report no change in p75/NGFR levels [86]. Furthermore, several lines of evidence demonstrate that the levels of basal forebrain NTRK1/NTRKA are reduced with aging with a concomitant increase in the ratio of p75/NGFR to NTRK1/NTRKA expression within the basal forebrain nuclei, which may be a very powerful inducer of neuronal degeneration [87].

In the African turquoise killifish brain, *NTRK1/NTRKA* and *p75/NGFR* are expressed in the ventral telencephalon, considering the homologous of the mammalian subpallium, caudal brainstem, in the periventricular grey zone of the OT and in the diffuse inferior lobe of the hypothalamus. In the brain of old animals, we observed a decreased number of p75/NGFR expressing cells and an unaltered number of positive NTRK1/NTRKA expressing cells during aging. This pattern denotes a region-specific expression of the two nerve growth factors receptors encoding genes, when compared to the neuroanatomical distribution of the neurotrophin family ligands analyzed in the adult brain of *N. furzeri* [10,11,12,13] Indeed NGF, BDNF, NT-4 and NT-6 are expressed in all regions of the adult brain, although with different patterns of expression. This raises the need of further investigations to explore the physiological role of neurotrophins and their receptors in the fish brain. Of relevance, interest should be addressed first to NGF and NT-6, which are considered as the specific ligand of *NTRK1/NTRKA* in teleosts.

*NTRK1/NTRKA* and *p75/NGFR* have been so far studied only in the developing brain of zebrafish. The expression of *NTRK1/NTRKA* appears 24 h post fertilization in the two domains of the cranial nerve ganglia flanking the hindbrain, in the spinal cord and in the rostral hindbrain [19]. *p75/NGFR* was expressed in the same cell populations as in mammals: in the cells of the ventral basal forebrain, in the midbrain and hindbrain nuclei, in cranial ganglia, in the region of the locus coeruleus, and in dorsal root ganglia. It was also detected at lower levels in the retina [38].

### 4.3. Nerve Growth Factor Receptors Are Not Expressed in Cholinergic Neurons of the Adult Brain of N. furzeri

Differently from the mammalian evidences, we were not able to observe any neuronal co-staining of *NTRK1/NTRKA*/ChaT nor *p75/NGFR*/ChaT. We observed some faint co-localization between ChaT neuronal projections and *ntrk1* expressing neurons, suggesting that cholinergic fibers contact *NTRK1/NTRKA* neurons. Neurotrophins and their receptors, along with the cholinergic system, represent an excellent example of conserved molecules throughout evolution, both at structural and physiological levels. However, it is likely that, due to the evolutionary history, killifish have evolved differentiated features of certain neuronal mechanisms. Notably, our results were mainly based on morphological observations, therefore we are designing *in vivo* experiments to better understand the physiological interactions between cholinergic system and neurotrophins in the brain of fish, as well as their roles during vertebrate aging.

## 5. Conclusions

Our results confirm that NGF receptors are evolutionary conserved in the African turquoise killifish, genes encoding *NTRK1/NTRKA* and *p75/NGFR* are not duplicate. Interestingly *NTRK1/NTRKA* is more abundantly expressed in the brain of young and old animals compared to *p75/NGFR,* but none of the two receptors is expressed in cholinergic neurons. This observation represents a novelty and crucial difference with reports from mammals, and more appropriate future studies are necessary to address this aspect.

## Supplementary Materials

The following are available online at https://www.mdpi.com/2076-3425/10/6/394/s1, Figure S1: Sense and antisense probe showing absence of signal in sense probe.
