# Supplementary material for: Cholinergic System and NGF Receptors: Insights from the Brain of the Short-Lived Fish Nothobranchius furzeri
Source: Brain Sci. 2020 Jun 20;10(6):394. doi: 10.3390/brainsci10060394 (PMC7348706; doi:10.3390/brainsci10060394)
Supplement: Supplementary file 1 [file brainsci-10-00394-s001.pdf]

Supplementary Materials: Cholinergic System and NGF Receptors: Insights from the Brain of the Short-Lived Fish *Nothobranchius furzeri*

Paolo de Girolamo [1,*], Adele Leggieri [1], Antonio Palladino [2], Carla Lucini [1], Chiara Attanasio [1] and Livia D'Angelo [1]

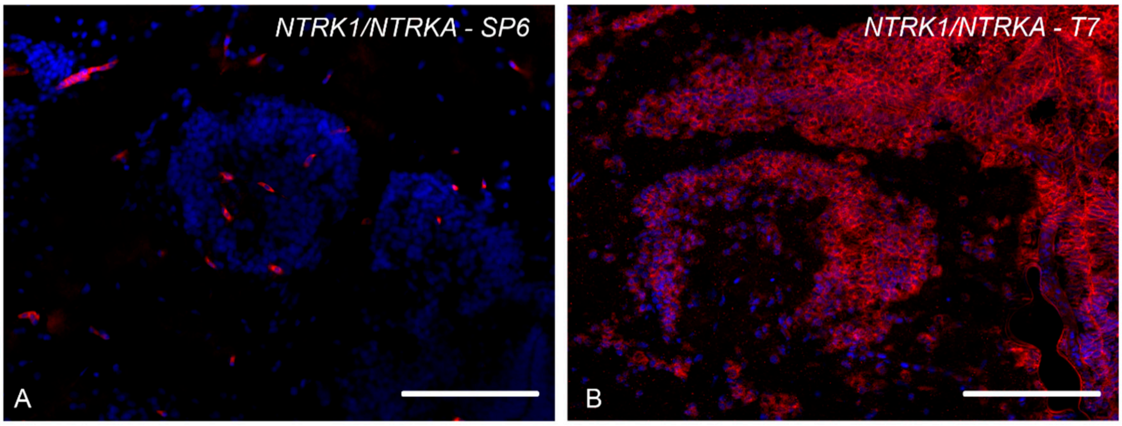

Figure S1: Sense and antisense probe showing the absence of signal in sense probe. Transverse section of the glomerular nucleus showing in (**A**) the absence of staining in the glomerular nucleus and diencephalon with the sense probe (SP6) against NTRK1/NTRKA in young animals; (**B**) the NTRK1/NTRKA labeling with antisense probe (T7) in the same brain area of young animals. Scale bar = 50μ.
